# PZLAST-MAG: full length protein sequence similarity search server of large-scale MAG proteins

**DOI:** 10.1093/bioadv/vbag129

**Published:** 2026-05-06

**Authors:** Koichi Higashi, Hitoshi Ishikawa, Ken Kurokawa, Hiroshi Mori

**Affiliations:** Department of Informatics, National Institute of Genetics, Mishima, Shizuoka 411-8540, Japan; ALIS, The Joint Support-Center for Data Science Research, Research Organization of Information and Systems, Minato-ku, Tokyo 105-6923, Japan; PEZY Computing, K. K., Chiyoda-ku, Tokyo 101-0052, Japan; Department of Informatics, National Institute of Genetics, Mishima, Shizuoka 411-8540, Japan; ALIS, The Joint Support-Center for Data Science Research, Research Organization of Information and Systems, Minato-ku, Tokyo 105-6923, Japan; Department of Informatics, National Institute of Genetics, Mishima, Shizuoka 411-8540, Japan

## Abstract

**Motivation:**

Metagenome-assembled genomes (MAGs) provide access to novel protein sequences from uncultured microbes, offering invaluable resources for studying protein diversity, structure prediction, and evolutionary analysis. However, despite the explosive growth of MAG-derived protein data, tools enabling fast and accurate similarity searches against large-scale MAG protein datasets remain limited.

**Results:**

We present PZLAST-MAG, a web server for ultra-fast sequence similarity searches against 0.4 billion MAG-derived protein sequences (0.1 trillion amino acids) from over 210 000 MAGs indexed in Microbiome Datahub. Implemented on PEZY-SC3 MIMD many-core processors, PZLAST-MAG achieves high accuracy and speed, with performance comparable to widely used tools such as DIAMOND and MMseqs2 based on our benchmark analyses. In addition to tabular alignments, PZLAST-MAG provides interactive visualizations of phylogenetic and environmental distributions and co-occurrence patterns of homologous proteins across MAGs. This combination enables rapid homolog mining of functionally important genes across diverse microbial lineages while simultaneously revealing their taxonomic and ecological contexts. Two use case analyses indicate its utility for homolog mining of metabolic enzyme genes and plasmid-derived genes.

**Availability and implementation:**

PZLAST-MAG is provided as a web-based service and is freely available at https://pzlast.nig.ac.jp/pzlast/mag without requiring registration.

## 1 Introduction

Metagenome-assembled genome (MAG) analysis has emerged as a powerful approach for obtaining genomic information of environmental microbes without cultivation (Quince *et al.* 2017). Genes reconstructed from MAGs are often full length, and the encoded proteins are likewise recovered as complete sequences. Such large-scale collections of environmental protein sequences are of particular importance for understanding the diversity of proteins in nature and are widely used as reference datasets for protein structure prediction and for evolutionary analyses of protein families (Jumper *et al.* 2021, Pavlopoulos *et al.* 2023). However, despite the growing demand, tools that allow fast similarity searches against large MAG-derived protein datasets remain limited. At present, the only widely available resources are MGnify Proteins Database (Gurbich *et al.* 2023, Richardson *et al.* 2023), which provides HMMER-based searches against proteins derived from MGnify metagenomic contigs, and MetaGraph, which enables k-mer-based searches across petabyte-scale DNA and protein sequence databases (Karasikov *et al.* 2025). This highlights the current lack of search platforms specifically designed for MAG proteins.

To address this, we previously compiled more than 210 000 MAGs from the public sequence repository of International Nucleotide Sequence Database Collaboration (INSDC), performed re-annotation of their genes and taxonomies, and organized associated environmental metadata using standardized ontologies. These data were released as the Microbiome Datahub, a publicly available MAG database (Mori *et al.* 2026, Microbiome Datahub 2026). The Microbiome Datahub currently provides access to 450 million MAG-derived protein sequences, each linked to taxonomic and environmental information. Compared with existing resources such as MGnify Protein Database, the Microbiome Datahub provides broader environmental coverage and longer protein sequences on average. Specifically, Microbiome Datahub integrates MAGs from 123 distinct environmental categories, whereas MGnify represents a more limited set of environments (18 categories). The definition of “environment” differs between databases. In MGnify, environmental categories are defined using a set of predefined labels (e.g. human gut, marine, soil, and various host-associated microbiomes such as cow rumen and chicken gut), many of which are associated with host-associated environments. In contrast, Microbiome Datahub adopts the Metagenome/Microbes Environmental Ontology (MEO), a controlled vocabulary that we developed to systematically describe microbial habitats. Each MAG is manually annotated using MEO terms, enabling more fine-grained and standardized classification across diverse environmental contexts. In addition, the average protein length in Microbiome Datahub (314.5 amino acids) is substantially longer than that in MGnify Protein (189.7 amino acids), reflecting the reconstruction of full-length genes from MAGs. Notably, only 32.9% of these proteins overlap with MGnify Protein sequences, meaning the majority represent unique proteins. The limited overlap can be explained by differences in environmental coverage, data sources (e.g. MAGs or metagenomic contigs), and gene prediction strategies. Given this extensive and unique protein collection, enabling sequence similarity searches against Microbiome Datahub MAG proteins would provide researchers with a powerful tool for exploring diverse proteins from a wide range of natural environments.

In this study, we extended our previously developed web server PZLAST (Mori *et al.* 2021, PZLAST web server 2026), originally designed for high-speed similarity searches against protein sequences predicted from short-read metagenomic data. In that framework, reference databases consist mainly of fragmented protein sequences, enabling efficient identification of homologs and their distribution across metagenomic samples. In contrast, PZLAST-MAG is designed to support large-scale similarity searches against nearly full-length protein sequences derived from MAGs. This shift from fragment-based to full-length protein databases enables more accurate identification of homologous sequences and facilitates analyses that require complete protein context. The new server, PZLAST-MAG, not only provides simple tabular output but also offers additional functionalities, including visualization of the phylogenetic and environmental distributions of homologous sequences, and analysis of homolog occurrence of multiple query proteins within the same MAG. In addition to sequence similarity, PZLAST-MAG enables interpretation of homologous proteins in an ecological context by mapping search results to environmental categories using MEO. By linking each MAG to standardized environmental annotations, users can examine the distribution of similar sequences across diverse habitats. This functionality facilitates ecological interpretation of protein functions and provides insights into the environmental contexts in which specific proteins are found.

## 2 Methods

### 2.1 PZLAST tool development

PZLAST-MAG is implemented on multiple PEZY-SC3 processors, which are many-core processors with a multiple-instruction, multiple-data (MIMD) architecture (Ishikawa *et al.* 2022). In an MIMD processor, each thread executes distinct instructions on different data, a feature well suited for handling the diverse computational stages of PZLAST-MAG. Rapid sequence similarity searches are achieved by distributing tasks in parallel across four PEZY-SC3 processors and exploiting the massive parallelism of each processor. Each PEZY-SC3 processor comprises 4096 cores with eight hardware threads per core, providing a total of 32 768 hardware threads per processor, which enables efficient parallel execution of sequence similarity searches. Compared to the previous PZLAST implementation on the PEZY-SC2 system, which required a specialized immersion-cooling environment, the current PEZY-SC3-based system adopts an air-cooled architecture. This transition allows deployment in standard server racks without specialized cooling infrastructure, significantly improving portability and practical usability. In addition, the PEZY-SC3 provides approximately twice the computational resources of PEZY-SC2, further enhancing throughput while retaining the core parallelization strategy of the original system.

The sequence similarity search algorithm of PZLAST-MAG is largely identical to that of the original PZLAST and is conceptually similar to BLASTP (Mori *et al.* 2021), employing a seed-and-extend strategy for fast and sensitive sequence similarity searches. To reduce the computational cost of the extension phase, PZLAST-MAG adopts a banded alignment approach, in which calculations are restricted to a limited region around each seed. In banded alignment, computational cost depends on the relative orientation of the sequences, and the total calculation area is minimized when the shorter sequence is assigned to the vertical axis and the longer sequence to the horizontal axis. In the original PZLAST, which primarily targeted short-read-derived protein sequences, this assignment was fixed. In contrast, PZLAST-MAG introduces an adaptive strategy that dynamically assigns the longer sequence to the horizontal axis and the shorter sequence to the vertical axis at runtime. This modification improves computational efficiency for MAG-derived protein datasets, where sequence lengths are highly variable.

Reference amino acid sequences for PZLAST-MAG were obtained from the Microbiome Datahub database. In the Microbiome Datahub pipeline, protein-coding genes are predicted from public MAG sequences deposited in INSDC using MetaGeneAnnotator (Noguchi *et al.* 2008). The resulting reference dataset comprises ∼143 GB of protein sequences (0.4 billion sequences totaling 0.1 trillion amino acids) derived from 214 427 MAGs (Microbiome Datahub protein sequence dataset 2026). In addition to protein sequences, the Microbiome Datahub provides GTDB-based taxonomic assignment results of genomic sequences and environmental annotations for each MAG based on the Metagenome and Microbes Environmental Ontology (MEO) (Chaumeil *et al.* 2022, Metagenome and Microbes Environmental Ontology 2026). Leveraging these annotations, PZLAST-MAG incorporates visualization modules implemented in JavaScript, which enable users to explore the phylogenetic and environmental distributions of homologous sequences identified by similarity searches.

### 2.2 Tool accuracy comparison

To evaluate the validity of PZLAST-MAG search results, we compared search precision and recall with those of two widely used tools for metagenomic analyses, DIAMOND and MMseqs2 (Steinegger and Söding 2017, Buchfink *et al.* 2021). For this benchmark, we used a subset of 10 000 000 sequences from the reference database. We employed 12 query proteins representing a range of sequence lengths (273–7746 amino acids), phylogenetic origins, conservation levels, and functional categories (Table 1, available as supplementary data at *Bioinformatics Advances* online). Precision and recall for the 10 000 000-sequence dataset were calculated using SSEARCH results (SSEARCH v36.3.8i: -s BL62 -E 1e-8 -m8) as the ground truth, since SSEARCH implements a rigorous Smith–Waterman alignment (Pearson 1991). For all tools, we used a consistent E-value threshold of 1e-8 and a maximum of 50 000 reported hits per query to ensure a fair comparison. To evaluate sensitivity across parameter settings, we tested multiple configurations for each tool. DIAMOND (v2.1.11): blastp was run in three modes: (i) --sensitive, (ii) --very-sensitive, and (iii) --ultra-sensitive, each with --evalue 1e-8 and --max-target-seqs 50 000. MMseqs2 (v18.8cc5c): easy-search was evaluated using two parameter settings: (i) -s 4.0 --start-sens 1 --sens-steps 1, and (ii) -s 6.0 --start-sens 2 --sens-steps 2, both with -e 1e-8 and --max-seqs 50 000. We note that an additional higher-sensitivity setting (-s 7.5), produced results identical to those obtained with -s 6.0 for the twelve query proteins. Therefore, we report results using -s 6.0 as a representative high-sensitivity configuration. Accuracy comparisons for SSEARCH, DIAMOND, and MMseqs2 were conducted on a Dell PowerEdge R660 server equipped with two Intel Xeon Gold 6430 2.1 GHz 32-core CPUs and 256 GB of RAM.

### 2.3 Use case analysis

As a first use case of PZLAST-MAG, we searched for two marker enzymes of the autotrophic CO_2_ fixation pathway, the dicarboxylate/4-hydroxybutyrate (DC/4HB) cycle: 4-hydroxybutyrate-CoA ligase and 4-hydroxybutyryl-CoA dehydratase (Huber *et al.* 2008, Kawashima *et al.* 2026). For this purpose, we used the protein sequences *from Ignicoccus hospitalis* strain KIN4/I (ABU81658.1 and ABU81777.1, respectively) as query inputs. To minimize non-specific hits, the search was conducted with an E-value threshold of <1e-100.

As a second use case, we analyzed plasmid-associated proteins from the human gut microbiome. Specifically, we selected two proteins, MobA and RepA (AAB49649.1 and AAB49650.1, respectively), encoded by the plasmid pBI143 (U30316.1), which has been reported to be globally distributed but enriched in human gut environments (Fogarty *et al.* 2024). Searches were performed with an E-value threshold of <1e-50 to identify high-confidence similar sequences.

## 3 Results

### 3.1 Accuracy comparison

**Figure 1:**
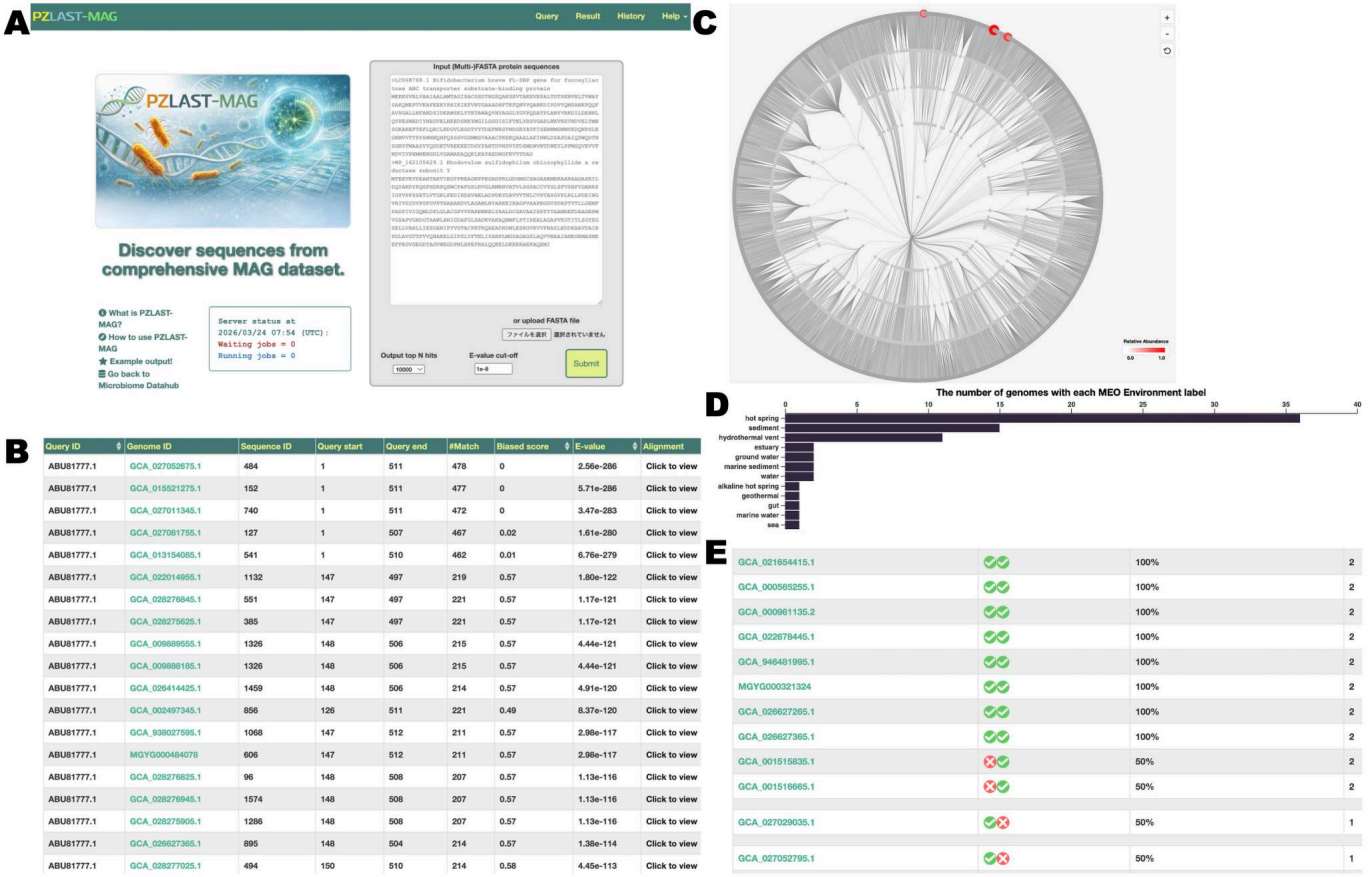
Overview of the PZLAST-MAG web interface and output visualization. (A) Top page of PZLAST-MAG, where users can submit up to 10 000 sequences per job. (B) Tabular results view, in which each row represents one hit and pairwise alignments can be view. (C) Phylogenetic tree distribution of hits, with red circles indicating the lineages from which the matched proteins are derived. (D) Environmental distribution of similar proteins based on MEO classes, shown as bar graphs. (E) Completion table summarizing the co-occurrence of homologous proteins from multiple queries within the same MAG.

The precision and recall rates of PZLAST-MAG and the two comparison tools are summarized in Table 1 and Figs 1 and 2, available as supplementary data at *Bioinformatics Advances* online. Overall, the performance of PZLAST-MAG was comparable to that of DIAMOND and MMseqs2 across most query proteins. Precision values were generally high for all tools, whereas recall values showed greater variability depending on the query. Notably, for longer query proteins (i.e. MbtE, RtxA, and PksD), MMseqs2 tended to show lower recall compared to the other tools (Fig. 2, available as supplementary data at *Bioinformatics Advances* online). This tendency was accompanied by a smaller number of detected hits (Table 1, available as supplementary data at *Bioinformatics Advances* online), suggesting reduced sensitivity for long and complex sequences under the tested conditions. One possible explanation is that heuristic pruning of MMSeqs2 during the search process may limit sensitivity for such queries. In contrast, for shorter and medium-length proteins, the differences among tools were less pronounced, and all methods showed broadly comparable performance. We note that the reference database used in this study (Microbiome Datahub) contains a large number of full-length proteins, including many long and multidomain sequences, which may influence tool performance depending on query characteristics. Taken together, these results indicate that PZLAST-MAG provides performance comparable to existing tools while maintaining stable sensitivity across a wide range of query protein lengths.

### 3.2 Tool usage

PZLAST-MAG is provided as a web-based service and is freely accessible at https://pzlast.nig.ac.jp/pzlast/mag without requiring registration. The current implementation is tightly coupled to a specialized many-core hardware architecture (PEZY-SC3), and therefore the standalone distribution of the software is not presently available. The server currently supports sequence similarity searches exclusively against the Microbiome Datahub protein database. A user can submit up to 10 000 sequences per job (Fig. 1A), with sequence lengths restricted to between 10 and 10 000 amino acids. Users can specify the maximum number of output hits (“Output top N hits”) and the E-value cutoff. Although PZLAST-MAG supports multiple job scheduling, the current system allows only one simultaneous job due to hardware resource constraints. Upon submission, a unique job ID is assigned, and the search typically requires ∼5–15 min to complete. The runtime of PZLAST-MAG depends on query size and sequence length. Longer query sequences require increased computation time; for example, a query using a large protein such as PksD (7746 amino acids) required ∼1 hour to complete. Results are then made available at a dedicated URL (e.g. https://pzlast.nig.ac.jp/pzlast/mag/result_info?job_id=example). By default, the top 10 000 hits per query are reported and summarized in multiple formats: (i) a tabular view (Fig. 1B), (ii) phylogenetic tree distributions (Fig. 1C), (iii) environmental distributions based on MEO classes (Fig. 1D), and (iv) a completion table summarizing the co-occurrence of homologous proteins from multiple queries within the same MAG (Fig. 1E). The tabular view provides pairwise alignment details and reference sequence information for each hit. The phylogenetic tree distribution summarizes the taxonomic placement of similar proteins within a prokaryotic phylogeny constructed from reference MAG sequences. The environmental distribution summarizes the number of MAGs from each environment, based on MEO classifications, that contain proteins similar to the query. By mapping search results to standardized environmental categories, this view provides an ecological perspective on the distribution of similar proteins across diverse habitats and helps identify environment-specific patterns. When multiple query sequences are submitted simultaneously, the completion table highlights their co-occurrence patterns across MAGs.

### 3.3 Use case analysis

The PZLAST-MAG search results for the two DC/4HB cycle marker enzymes are shown in Fig. 1B–E. The DC/4HB pathway is known to be present in certain chemolithoautotrophic members of the archaeal phylum Thermoproteota (Garritano *et al.* 2022). Using an E-value threshold of <1e-100, PZLAST-MAG identified 49 hits for 4-hydroxybutyrate-CoA ligase and 68 hits for 4-hydroxybutyryl-CoA dehydratase. Most of the corresponding MAGs belonged to Thermoproteota (Fig. 1C; two red circles at the upper right); however, a subset of MAGs containing 4-hydroxybutyryl-CoA dehydratase were derived from the archaeal phylum Asgardarchaeota (Fig. 1C; red circle at the upper center). Proteins similar to both enzymes are predominantly found in hot springs, hydrothermal vents, and marine sediments, supporting the notion that the DC/4HB pathway plays an important role in autotrophic, low-carbon environments (Fig. 1D). As shown in the completion analysis (Fig. 1E), Asgardarchaeota-derived MAGs contained 4-hydroxybutyryl-CoA dehydratase but lacked 4-hydroxybutyrate-CoA ligase, suggesting that the complete DC/4HB cycle is not functional in this lineage. Comparative genomic analyses have reported that several MAGs from Asgardarchaeota contain most of the genes required for the DC/4HB cycle (Garritano *et al.* 2022), suggesting that this lineage may employ a modified version of the DC/4HB cycle, although experimental evidence is not yet available.

As a second use case, we analyzed plasmid-associated proteins (MobA and RepA) derived from the human gut-associated plasmid pBI143 (Fogarty *et al.* 2024). Using an E-value threshold of <1e-50, PZLAST-MAG identified sequences similar to these proteins across the reference MAG dataset. The environmental distribution of the resulting hits (Fig. 3, available as supplementary data at *Bioinformatics Advances* online) showed strong enrichment in fecal-associated environments, consistent with previous reports that pBI143 is predominantly found in the human gut microbiome. In total, ∼110 MAGs were identified as containing sequences similar to MobA and RepA. Although plasmids are generally underrepresented in MAG datasets due to differences in sequence composition (e.g. tetranucleotide frequency) and challenges in genome assembly and binning (Maguire *et al.* 2020), these results indicate that PZLAST-MAG can recover plasmid-associated sequences to a meaningful extent. This demonstrates the applicability of the PZLAST-MAG not only to chromosomally encoded genes but also to mobile genetic elements.

## 4 Conclusion

PZLAST-MAG enables ultra-fast and highly accurate sequence similarity searches against public MAG-derived protein sequences. Beyond returning homolog hits, the web server integrates downstream analyses and visualization: users can inspect the phylogenetic distribution of hits across lineages, the environmental distribution of source MAGs, and the co-occurrence of multiple query proteins within the same MAGs. This combination enables rapid homolog mining of functionally important genes across diverse microbial lineages while simultaneously revealing their taxonomic and ecological contexts. The reference database of PZLAST-MAG is based on Microbiome Datahub and will be updated in conjunction with future releases of that resource. We plan to perform database updates on an annual basis, with versioning information provided to ensure reproducibility of analyses.

## Supplementary Material

vbag129_Supplementary_Data

## Data Availability

PZLAST-MAG is provided as a web-based service and is freely available at https://pzlast.nig.ac.jp/pzlast/mag without requiring registration.
