# Supplementary material for: PZLAST-MAG: full length protein sequence similarity search server of large-scale MAG proteins
Source: Bioinform Adv. 2026 May 6;6(1):vbag129. doi: 10.1093/bioadv/vbag129 (PMC13189709; doi:10.1093/bioadv/vbag129)

Supplementary Figure S1. Distribution of precision and recall across tools and parameter settings.

Box plots showing the distribution of precision (left) and recall (right) across twelve query proteins for each tool and parameter setting. Results are shown for MMseqs2 (two parameter settings: MMSeqs2_s4 = MMSeqs2 -s 4.0, MMSeqs2_s6 = MMSeqs2 -s 6.0), DIAMOND (three sensitivity modes: DIAMOND_s = DIAMOND --sensitive, DIAMOND_vs = DIAMOND --very-sensitive, DIAMOND_uls = DIAMOND --ultra-sensitive), and PZLAST-MAG. Box plots summarize the overall distribution.

Supplementary Figure S2. Per-protein performance comparison across tools and parameter settings.

Heatmaps showing precision (left) and recall (right) for each query protein across different tools and parameter settings. Rows represent individual query proteins with varying sequence lengths and functional categories, and columns represent tools (MMseqs2, DIAMOND, and PZLAST-MAG). Color intensity indicates performance values (%). This visualization highlights variability in performance depending on query characteristics.

Supplementary Figure S3. Environmental distribution of sequences similar to plasmid-associated proteins (MobA and RepA) by PZLAST-MAG.

Bar plots showing the number of MAGs containing sequences similar to MobA (top) and RepA (bottom) across environmental categories defined by MEO. Both proteins exhibit strong enrichment in fecal-associated environments, consistent with their reported association with the human gut microbiome.

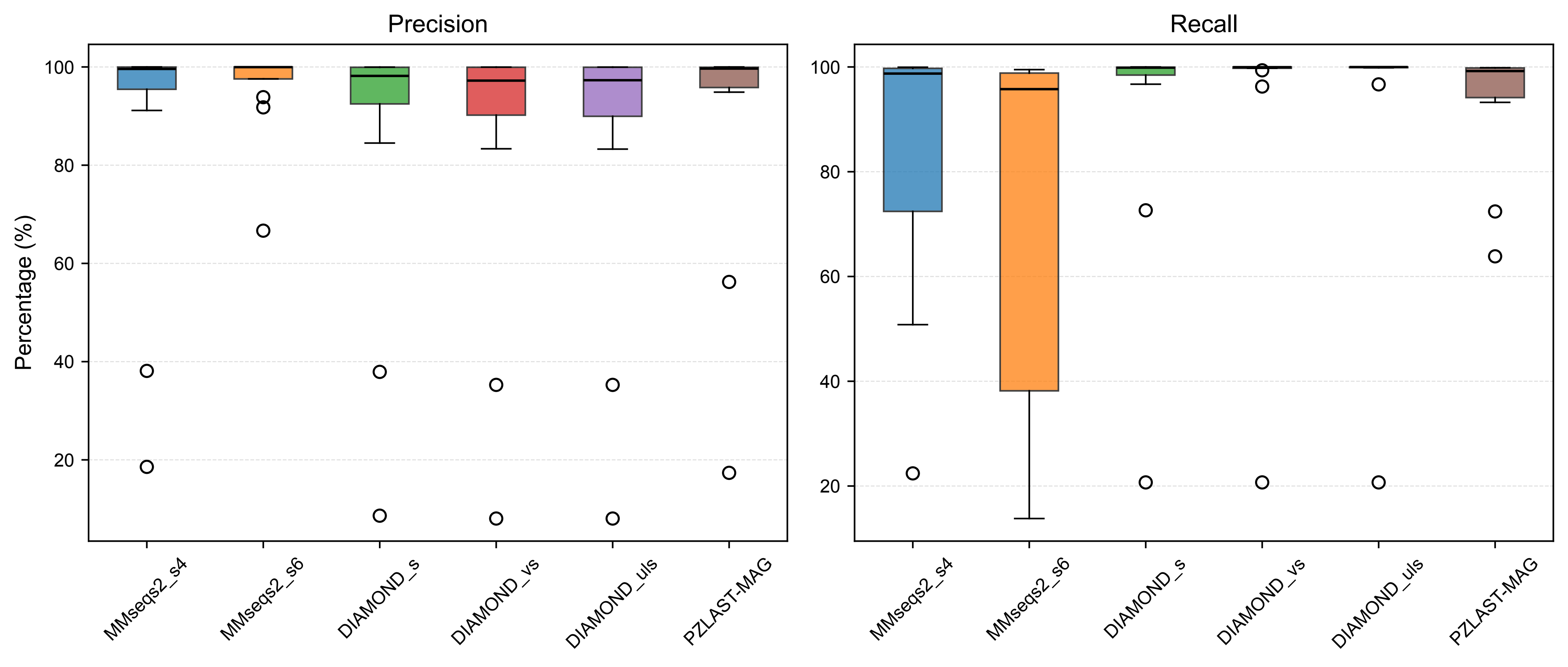

Precision
Recall
Percentage (%)
100
80
60
40
20
MMseqs2_s4
MMseqs2_s6
DIAMOND_s
DIAMOND_vs
DIAMOND_uls
PZLAST-MAG

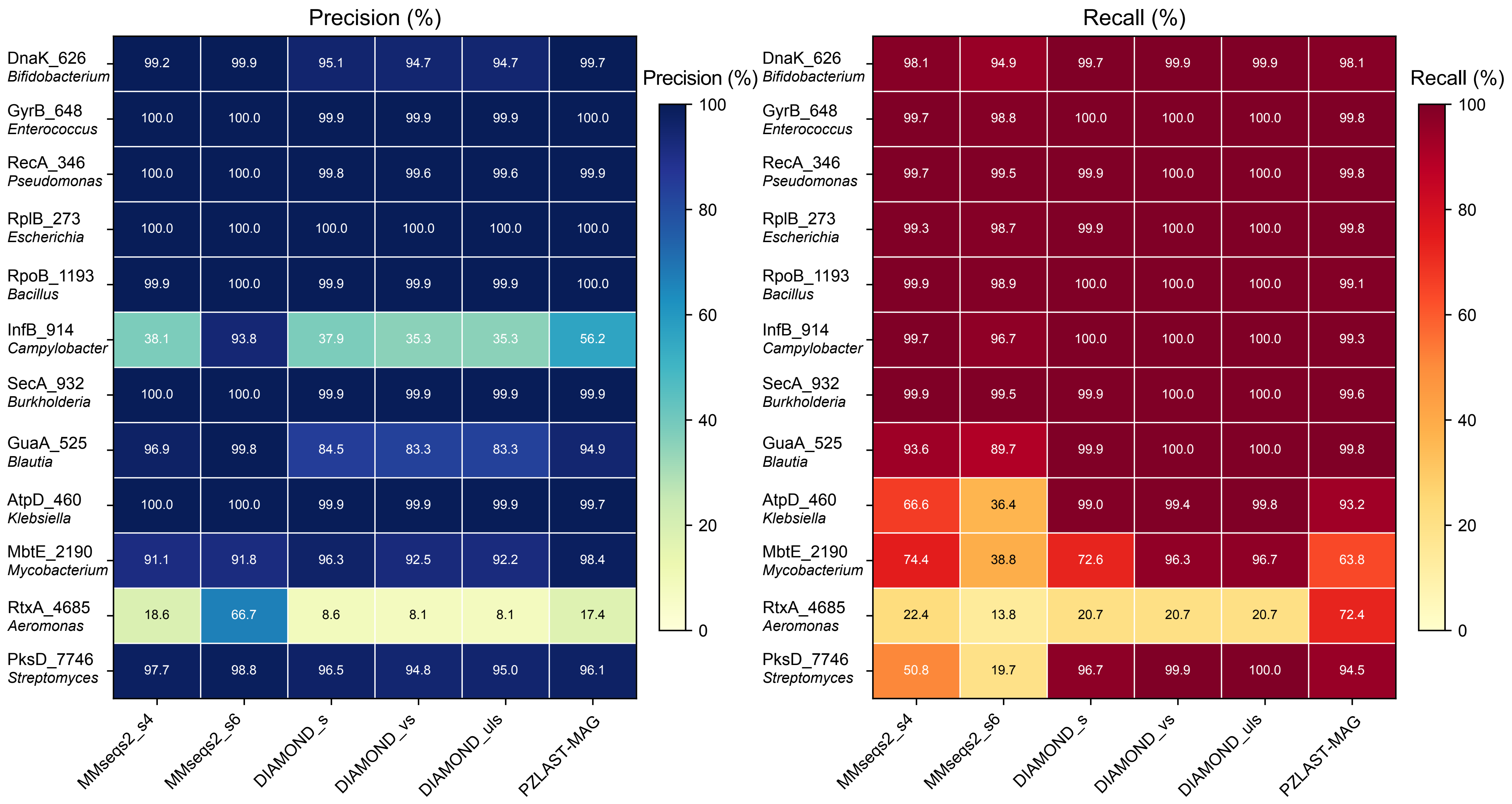

Precision (%)
DnaK_626 Bifidobacterium
GyrB_648 Enterococcus
RecA_346 Pseudomonas
RplB_273 Escherichia
RpoB_1193 Bacillus
InfB_914 Campylobacter
SecA_932 Burkholderia
GuaA_525 Blautia
AtpD_460 Klebsiella
MbtE_2190 Mycobacterium
RtxA_4685 Aeromonas
PksD_7746 Streptomyces
99.2 99.9 95.1 94.7 94.7 99.7
100.0 100.0 99.9 99.9 99.9 100.0
100.0 100.0 99.8 99.6 99.6 99.9
100.0 100.0 100.0 100.0 100.0 100.0
99.9 100.0 99.9 99.9 99.9 100.0
38.1 93.8 37.9 35.3 35.3 56.2
100.0 100.0 99.9 99.9 99.9 99.9
96.9 99.8 84.5 83.3 83.3 94.9
100.0 100.0 99.9 99.9 99.9 99.7
91.1 91.8 96.3 92.5 92.2 98.4
18.6 66.7 8.6 8.1 8.1 17.4
97.7 98.8 96.5 94.8 95.0 96.1
MMseqs2_s4
MMseqs2_s6
DIAMOND_s
DIAMOND_vs
DIAMOND_uls
PZLAST-MAG
Precision (%)
100
80
60
40
20
0
Recall (%)
DnaK_626 Bifidobacterium
GyrB_648 Enterococcus
RecA_346 Pseudomonas
RplB_273 Escherichia
RpoB_1193 Bacillus
InfB_914 Campylobacter
SecA_932 Burkholderia
GuaA_525 Blautia
AtpD_460 Klebsiella
MbtE_2190 Mycobacterium
RtxA_4685 Aeromonas
PksD_7746 Streptomyces
98.1 94.9 99.7 99.9 99.9 98.1
99.7 98.8 100.0 100.0 100.0 99.8
99.7 99.5 99.9 100.0 100.0 99.8
99.3 98.7 99.9 100.0 100.0 99.8
99.9 98.9 100.0 100.0 100.0 99.1
99.7 96.7 100.0 100.0 100.0 99.3
99.9 99.5 99.9 100.0 100.0 99.6
93.6 89.7 99.9 100.0 100.0 99.8
66.6 36.4 99.0 99.4 99.8 93.2
74.4 38.8 72.6 96.3 96.7 63.8
22.4 13.8 20.7 20.7 20.7 72.4
50.8 19.7 96.7 99.9 100.0 94.5
MMseqs2_s4
MMseqs2_s6
DIAMOND_s
DIAMOND_vs
DIAMOND_uls
PZLAST-MAG
Recall (%)
100
80
60
40
20
0

Showing results of MobA

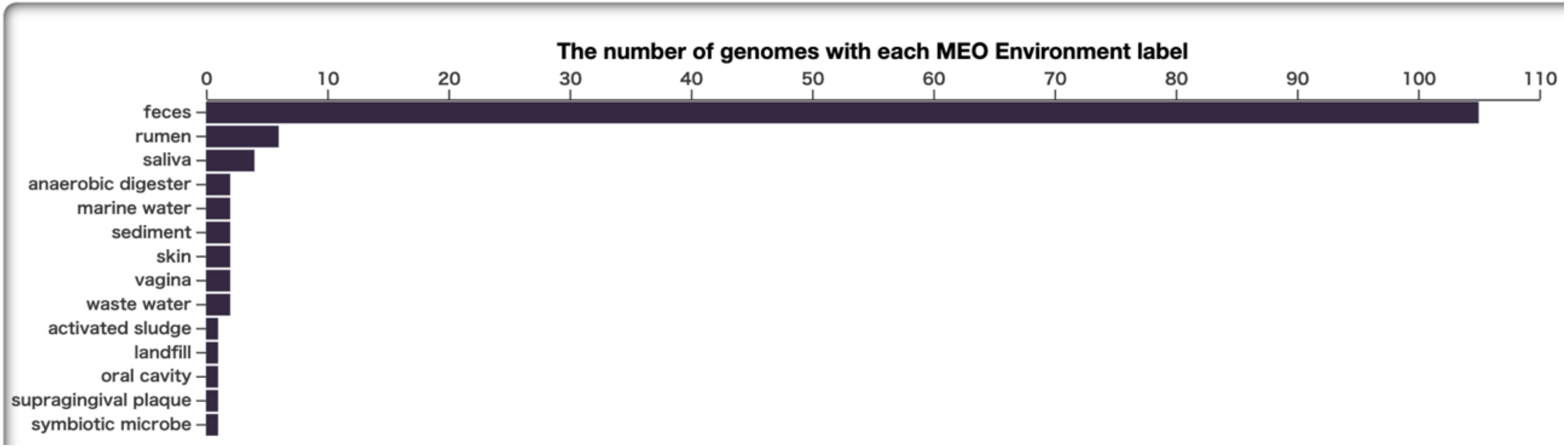

Showing results of RepA

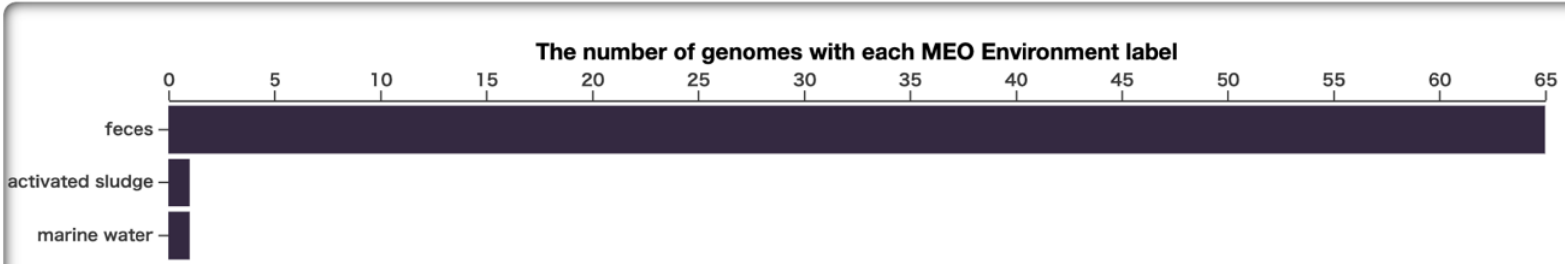

Supplement: vbag129_Supplementary_Data [file vbag129_supplementary_data.zip › SupplementaryFiguresS1-S3.pdf]
